# Levodopa Carbidopa Intestinal Gel in Advanced Parkinson’s Disease: DUOGLOBE Final 3-Year Results

**DOI:** 10.3233/JPD-225105

**Published:** 2023-07-25

**Authors:** K. Ray Chaudhuri, Norbert Kovács, Francesco E. Pontieri, Jason Aldred, Paul Bourgeois, Thomas L. Davis, Esther Cubo, Marieta Anca-Herschkovitsch, Robert Iansek, Mustafa S. Siddiqui, Mihaela Simu, Lars Bergmann, Mayra Ballina, Pavnit Kukreja, Omar Ladhani, Jia Jia, David G. Standaert

**Affiliations:** a Parkinson’s Foundation International Centre of Excellence, King’s College Hospital, and King’s College Institute of Psychiatry, Biomedical Research Centre, Psychology & Neuroscience, London, United Kingdom; b Department of Neurology, University of Pécs, Pécs, Hungary; c Department of Neuroscience, Mental Health and Sensory Organs, Sapienza University of Rome, Rome, Italy; d Santa Lucia Foundation, IRCCS, Rome, Italy; e Selkirk Neurology, Spokane, WA, USA; f Department of Neurology AZ Groeninge, Kortrijk, Belgium; g Department of Neurology, Vanderbilt University Medical Center, Nashville, TN, USA; h Neurology Department, Hospital Universitario Burgos, Burgos, Spain; i Department of Neurology, Edith Wolfson Medical Center, Holon, Israel; j Kingston Centre, Monash Health, Melbourne, Victoria, Australia; k Department of Neurology, Wake Forest School of Medicine, Winston Salem, NC, USA; l Department of Neurology, Victor Babes Universityof Medicine and Pharmacy, Timisoara, Romania; m AbbVie Inc., North Chicago, IL, USA; n Department of Neurology, University of Alabama at Birmingham, Birmingham, AL, USA

**Keywords:** DUOGLOBE, Parkinson’s disease, levodopa-carbidopa intestinal gel, dyskinesia, real-world data

## Abstract

**Background:**

Levodopa-carbidopa intestinal gel (LCIG) improves motor and non-motor symptoms in patients with advanced Parkinson’s disease (aPD).

**Objective:**

To present the final 36-month efficacy and safety results from DUOGLOBE (DUOdopa/Duopa in Patients with Advanced Parkinson’s Disease – a GLobal OBservational Study Evaluating Long-Term Effectiveness; NCT02611713).

**Methods:**

DUOGLOBE was an international, prospective, long-term, real-world, observational study of patients with aPD initiating LCIG in routine clinical care. The primary endpoint was change in patient-reported “Off” time to Month 36. Safety was assessed by monitoring serious adverse events (SAEs).

**Results:**

Significant improvements in “Off” time were maintained over 3 years (mean [SD]: –3.3 hours [3.7]; *p* < 0.001). There were significant improvements to Month 36 in total scores of the Unified Dyskinesia Rating Scale (–5.9 [23.7]; *p* = 0.044), Non-Motor Symptoms Scale (–14.3 [40.5]; *p* = 0.002), Parkinson’s Disease Sleep Scale-2 (–5.8 [12.9]; *p* < 0.001), and Epworth Sleepiness Scale (–1.8 [6.0]; *p* = 0.008). Health-related quality of life and caregiver burden significantly improved through Months 24 and 30, respectively (Month 24, 8-item Parkinson’s Disease Questionnaire Summary Index, –6.0 [22.5]; *p* = 0.006; Month 30, Modified Caregiver Strain Index, –2.3 [7.6]; *p* = 0.026). Safety was consistent with the well-established LCIG profile (SAEs: 54.9% of patients; discontinuations: 54.4%; discontinuations due to an adverse event: 27.2%). Of 106 study discontinuations, 32 patients (30.2%) continued LCIG outside the study.

**Conclusion:**

DUOGLOBE demonstrates real-world, long-term, reductions in motor and non-motor symptoms in patients with aPD treated with LCIG.

## INTRODUCTION

Levodopa is considered the “gold standard” for the treatment of Parkinson’s disease (PD) [1]. With disease progression, the benefit of oral levodopa diminishes as the therapeutic window narrows due to the short half-life of levodopa and progressive denervation of the striatum and subsequent postsynaptic plasticity [2]. In addition, erratic gastric emptying (a common symptom with advancing PD) leads to irregular gastrointestinal absorption of oral levodopa and unstable plasma levodopa concentrations, collectively resulting in pulsatile stimulation [2, 3]. Thus, motor and non-motor symptoms become increasingly difficult to manage with oral levodopa, which can cause patients to experience predictable and unpredictable fluctuations between “On” periods with potentially disabling dyskinesias and “Off” periods when the patient experiences a return of their parkinsonian symptoms and may even be “frozen” and akinetic [2, 4, 5]. These symptoms progressively worsen over time and greatly impact patients’ functional capacity and health-related quality of life (HRQoL) [6, 7].

Levodopa carbidopa intestinal gel (LCIG; also known as carbidopa-levodopa enteral suspension [CLES]) is a stable gel suspension of levodopa-carbidopa (20 mg/mL and 5 mg/mL, respectively) for continuous daytime infusion in patients with advanced PD (aPD) [8, 9]. Continuous infusion enables levodopa concentrations to be kept at a constant level within the individual’s optimal therapeutic window, making LCIG a meaningful option for managing aPD [3, 10]. Results from controlled clinical trials have demonstrated beneficial effects of LCIG therapy on motor symptoms, including reductions in “Off” time, increases in “On” time without troublesome dyskinesia, and improvements in HRQoL and activities of daily living [11–14]. Notably, results from a recent randomized clinical trial demonstrated an improved reduction in dyskinesia as measured by the Unified Dyskinesia Rating Scale (UDysRS) following treatment with LCIG vs. optimized medical treatment [15]. LCIG has also demonstrated beneficial effects on motor and non-motor symptoms in observational studies [16–21], and systematic literature reviews and meta-analyses [22, 23].

DUOdopa/Duopa in Patients with Advanced Parkinson’s Disease, a GLobal OBservational Study Evaluating Long-Term Effectiveness (DUOGLOBE), was the first international, fully prospective, long-term, non-interventional, post-marketing, observational study of patients with aPD treated with LCIG in a routine clinical setting. One-year interim results indicated significant improvements in motor symptoms (including “Off” time and dyskinesia), non-motor symptoms (including sleep), HRQoL, and caregiver burden, with safety events consistent with those noted in previous controlled clinical trials and observational studies [24]. This report presents the final 36-month results from the DUOGLOBE study.

## Methods

### Study design and treatment

DUOGLOBE was a global multicenter, single-arm, non-interventional, post-marketing, observational study (NCT02611713) conducted in 55 sites across 10 countries (Australia, Belgium, Hungary, Israel, Italy, Romania, Slovenia, Spain, United Kingdom, and the United States) [24].

Detailed methods for this study have been published [24]. In brief, patients enrolled in this 36-month real-world study had aPD for whom their physicians decided to start LCIG treatment according to the local product label and specific reimbursement criteria. LCIG dosage was individually optimized for clinical response and concomitant PD medications were permitted at the discretion of the treating physician. National and/or local independent ethics committees, institutional review boards, and/or health authorities in all countries approved the protocol, patient information, and informed consent requirements according to the applicable national regulatory requirements.

### Patients

In addition to patient inclusion being based on local LCIG label and reimbursement criteria, key eligibility criteria included patients having no prior exposure to LCIG; a Mini-Mental State Examination score≥24; no prior surgery for PD including, but not limited to, deep brain stimulation or cell transplantation (for non-US centers); and no current subcutaneous apomorphine infusion (with≥4 weeks required between drug discontinuation and study inclusion). All patients and caregivers provided informed consent.

### Assessments

Assessment occurred before starting LCIG (baseline) therapy; at Day 1 (start of LCIG treatment via percutaneous endoscopic gastrostomy with jejunal extension for those patients who participated in the preceding nasojejunal test phase only); and at routinely scheduled visits, which were closest to Months 3, 6, 12, 18, 24, 30, and 36 (±14 days each), or at the time of premature discontinuation.

The primary endpoint was the change in the number of hours of “Off” time as reported by the patient for the day before the clinical visit (baseline) compared with the same measure at Month 36. Secondary endpoints included mean change from baseline to the end of the study in the Unified Parkinson’s Disease Rating Scale (UPDRS) Part II (activities of daily living); Part III (motor examination performed in the “On” state); and the following items in Part IV: item 33 (dyskinesia-related disability), item 34 (dyskinesia-related pain), and item 35 (early morning dystonia). Secondary endpoints also included the UDysRS total score (signs/symptoms of dyskinesia) and subdomain scores, Non-motor Symptom Scale (NMSS) total and subdomain scores, Parkinson’s Disease Sleep Scale-2 (PDSS-2) total score (sleep quality), Epworth Sleepiness Scale (ESS) total score (daytime somnolence), 8-item PD Questionnaire (PDQ-8) summary index (HRQoL), and caregiver burden (Modified Caregiver Strain Index).

Only serious adverse events (SAEs) and adverse events (AEs) leading to premature discontinuation were reported as part of this observational study from initiation of LCIG treatment to 30 days after the last study visit.

### Statistical analysis

Planned enrollment was approximately 200 patients. It was assumed that 60% of patients would complete the 36-month follow-up period and that the mean (SD) decrease from baseline to Month 36 in the number of hours in “Off” time would be 4 hours. Therefore, the distance from the lower limit of the 95% confidence interval (CI) to the mean decrease would be 0.72 hours (i.e., the lower limit of the 95% CI of the mean decrease from baseline to Month 36 would be 3.28 hours). Significance for all efficacy measures was determined using a one-sample *t* test compared with baseline efficacy assessments. Safety assessments were performed with the safety population, which included all patients who had nasojejunal and/or percutaneous endoscopic gastrostomy with jejunal extension placement, irrespective of whether patients withdrew prematurely or not. Efficacy assessments were performed with the full analysis population, which included all patients in the safety population who had at least one post-baseline effectiveness assessment after undergoing percutaneous endoscopic gastrostomy with jejunal extension placement.

### Data sharing

Clinical trial data can be requested by any qualified researchers who engage in rigorous, independent scientific research, and will be provided following review and approval of a research proposal and statistical analysis plan and execution of a data sharing agreement. Data requests can be submitted at any time and the data will be accessible for 12 months, with possible extensions considered. For more information on the process, or to submit a request, visit the following link: https://www.abbvieclinicaltrials.com/hcp/data-sharing/.

## Results

### Patients

Patient demographics and baseline characteristics of the 195 patients included in the analysis have been reported previously [24]. Most patients were male (61.5%), mean (SD) age was 70.2 (8.2) years, and mean duration of PD was 11.2 (4.8) years (Table 1). Two patients previously underwent deep brain stimulation. Mean (SD) LCIG treatment duration was 923.5 (367.2) days, with a median daily duration of LCIG infusion of 16 hours from Day 1 through Month 36. At each timepoint, between nine and 12 patients were receiving 24-hour LCIG therapy.

**Table 1:** Baseline demographics and clinical characteristics

Characteristic	Total
	*N* = 195
Sex, *n* (%)	
Male	120 (61.5)
Female	75 (38.5)
Age (y); mean±SD	70.2±8.2
<65 y, *n* (%)	44 (22.6)
65–75 y, *n* (%)	95 (48.7)
>75 y, *n* (%)	56 (28.7)
BMI; mean±SD BMI, kg/m^2^	25.9±4.1^a^
PD duration, y: mean±SD	11.2±4.8
<10 y, *n* (%)	94 (48.5)
≥10 y, *n* (%)	100 (51.5)
Time to LCIG initiation, y; mean±SD from:	
PD symptoms	12.2±5.0
Start of motor fluctuations	5.6±4.7
MMSE total score^b^; mean±SD	27.7±2.2
Hoehn and Yahr stage; *n* (%)	
During “On”	
1	4 (2.1)
1.5	0
2	33 (17.6)
2.5	21 (11.2)
3	80 (42.6)
4	43 (22.9)
5	7 (3.7)
Missing	7
During “Off”	
1	0
1.5	2 (1.1)
2	6 (3.2)
2.5	15 (8.1)
3	59 (31.7)
4	82 (44.1)
5	22 (11.8)
Missing	9
Daily “Off” time (h); mean±SD	6.0±3.4
UPDRS Part II (ADL); mean±SD	14.8±7.8
UPDRS Part III (motor function); mean±SD	27.6±13.2
UDysRS total score; mean±SD	33.7±21.1
NMSS total score; mean±SD	88.2±51.1
PDSS-2 total score (sleep quality); mean±SD	26.6±11.7
ESS total score (daytime sleepiness); mean±SD	9.8±5.3
PDQ-8 summary index (HRQoL); mean±SD	45.1±18.1
MCSI total score (caregiver burden); mean±SD	10.9±6.4

^a^*n*=182. ^b^Patient MMSE total score at baseline must be≥24 for inclusion. ADL, activities of daily living; BMI, body mass index; ESS, Epworth Sleepiness Scale; HRQoL, health-related quality of life; LCIG, levodopa-carbidopa intestinal gel; MCSI, Modified Caregiver Strain Index; MMSE, Mini-Mental State Examination; NMSS, Non-Motor Symptoms Scale; PD, Parkinson’s disease; PDSS-2, Parkinson’s Disease Sleep Scale-2; PDQ-8, 8-item Parkinson’s Disease Questionnaire; SD, standard deviation; UDysRS, Unified Dyskinesia Rating Scale, UPDRS, Unified Parkinson’s Disease Rating Scale.

**Fig. 1:**
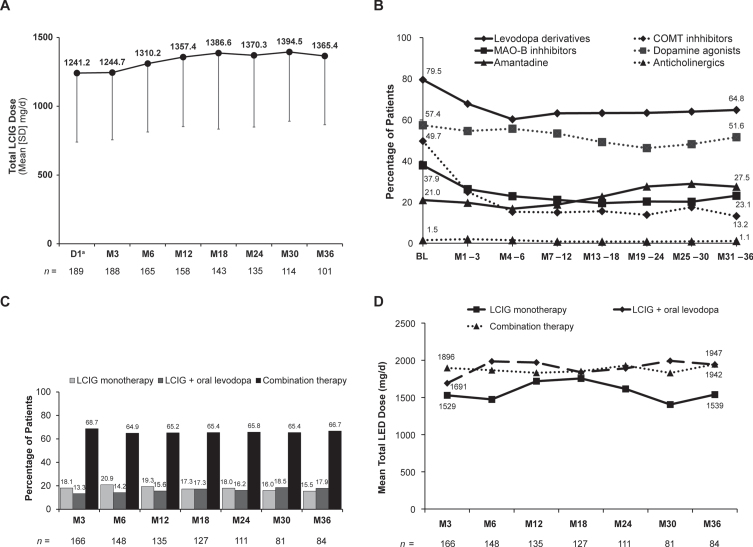
Stability of therapy over time. (A) LCIG dose, (B) anti-PD comedications, (C) monotherapy vs. combination therapy, and (D) total levodopa equivalent dose. ^a^Only patients who participated in the preceding nasojejunal test phase were assessed at D1. BL, baseline; COMT, catechol-O-methyltransferase; D, day; LCIG, levodopa-carbidopa intestinal gel; LED, levodopa equivalent dose; M, month; MAO-B, monoamine oxidase-B; PD, Parkinson’s disease; SD, standard deviation.

Of note, mean (SD) daily dose of LCIG remained fairly stable from Day 1 (1241.2 [501.6] mg/day) through Month 36 (1365.4 [499.1] mg/day) (Fig. 1A). Within the first 6 months of LCIG use, there were decreases in concomitant use of oral levodopa derivatives; monoamine oxidase B inhibitors; and, most prominently, catechol-O-methyltransferase inhibitors that remained steady throughout the next 2.5 years (Fig. 1B). At Month 3, 18.1% of patients were receiving LCIG monotherapy and 13.3% were receiving LCIG in combination with oral levodopa only (“levodopa monotherapy”); these percentages remained relatively stable throughout the study (Month 36 : 15.5% and 17.9%, respectively) (Fig. 1C). Total levodopa equivalent dose also remained stable throughout the study, regardless of whether patients were receiving LCIG as a monotherapy or in combination with other PD medications (Fig. 1D).

Of 195 enrolled patients, 106 (54.4%) discontinued the study prematurely with AEs being the primary reason (patients could have multiple reasons for discontinuation) in 48 of these patients (Supplementary Figures 1 and 2). Importantly, of the 106 patients who discontinued the study, 32 (30.2%) continued treatment with LCIG outside the study (Supplementary Figure 2).

### Motor complications

**Fig. 2:**
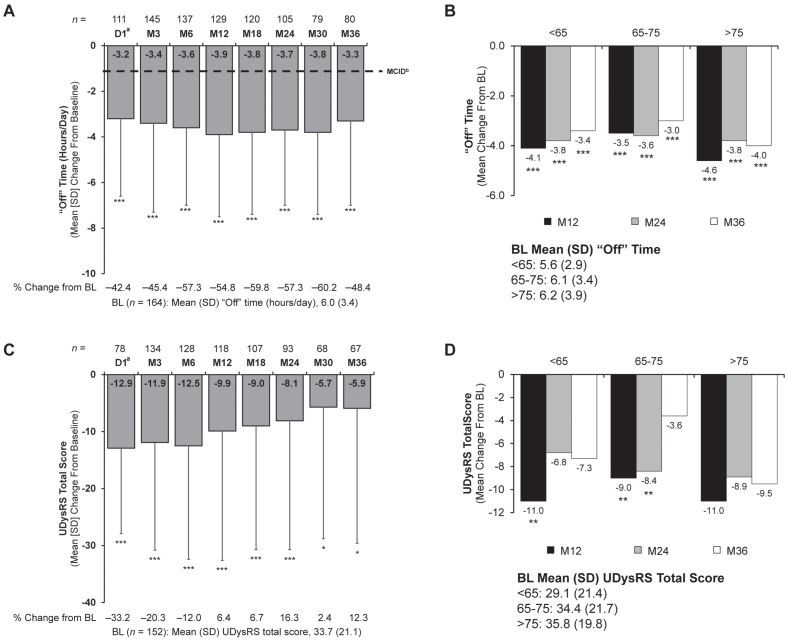
Change from baseline to Month 36 in (A) patient reported “Off” time, (B) “Off” time by age subgroups, (C) dyskinesia as measured by UDysRS total score, and (D) UDysRS total score by age subgroups. Significance level for change from baseline was determined using the one-sample *t* test. *p<0.05; **p<0.01; ***p<0.001. ^a^Only patients who participated in the preceding nasojejunal test phase were assessed at D1. ^b^As reported in Hauser et al [26]. BL, baseline; D, day; LCIG, levodopa-carbidopa intestinal gel; M, month; MCID, minimal clinically important difference; SD, standard deviation; UDysRS, Unified Dyskinesia Rating Scale.

Mean (SD) daily hours spent in the “Off” state significantly decreased from baseline to Day 1 and remained decreased through Month 36 (Month 36 [primary endpoint]: –3.3 [3.7]; *p* < 0.001) (Fig. 2A). Changes from baseline at Month 36 were statistically significant in all age groups (Fig. 2B).

For UDysRS total scores, significant reductions from baseline were observed at all time points through Month 36 (Month 36 mean [SD]: –5.9 [23.7]; *p* = 0.044) (Fig. 2C). Exploratory analysis was conducted to evaluate the effect of LCIG on UDysRS at Month 36 based on age; there was no significant difference at any of the ages evaluated (Fig. 2D). The composite historical score was significantly reduced from baseline through Month 36 (Month 36 *p* = 0.012). The subdomain of “On” dyskinesia (Part I) was significantly reduced from baseline until Month 24 (Month 24 *p* = 0.001), with “Off” dystonia (Part II) significantly reduced through Month 36 (Month 36 *p* < 0.001); both these improvements exceeded the level of clinical relevance [25]. Significant reductions from baseline during the study were seen for other components of the UDysRS, including the objective score through Month 18 (Month 18 *p* = 0.045), the impairment subdomain (Part III) through Month 6 (Month 6 *p* < 0.001), and the disability subdomain (Part IV) through Month 24 (Month 24 *p* = 0.033) (Supplementary Figure 3). Additional support for the reduction of the presence and symptoms of dyskinesia and dystonia through Month 36 was seen in the UPDRS scale, including dyskinesia-related disability (mean [SD], –0.4 [1.3]; *p* = 0.016), dyskinesia-related pain (–0.4 [1.0]; *p* < 0.001), and early morning dystonia (–0.2 [0.6]; *p* < 0.001) (Supplementary Figure 4).

UPDRS Part II and III scores, after initial improvements (significant for UPDRS III until Month 3), demonstrated significant worsening from baseline to Month 36 in the overall group (Supplementary Figure 4). Patients aged≥65 years showed significant worsening in UPDRS Part II and III scores at Month 36, while patients aged younger than 65 years had nominal, but not statistically significant, improvements in UPDRS Part III scores throughout the study (Supplementary Figure 4).

A summary of baseline and change-from-baseline values for patients with Month 36 data can be found in Table 2.

**Table 2:** Change from baseline to month 36 in motor and non-motor endpoints among patients with 36-month data

Endpoints	*n*	Baseline	Change from baseline
Daily “Off” time (h)	80	5.8 (3.1)	–3.3 (3.7)***
UDysRS total score	67	35.2 (21.4)	–5.9 (23.7)*
UPDRS Part II (ADL)	85	13.4 (8.5)	4.3 (8.3)***^a^
UPDRS Part III (motor function)	83	24.9 (13.6)	5.8 (13.9)***^a^
NMSS total score	79	83.7 (46.5)	–14.3 (40.5)**
PDSS-2 total score (sleep quality)	85	27.7 (12.3)	–5.8 (12.9)***
ESS total score (daytime sleepiness)	84	9.6 (5.3)	–1.8 (6.0)**
PDQ-8 summary index (HRQoL)	84	45.2 (18.6)	–2.5 (19.6)
MCSI total score (caregiver burden)	52	12.3 (6.8)	–1.3 (7.8)

Table includes patients with Month 36 data only. All data are presented as mean±SD. ^a^Reflects worsening from baseline. *p<0.05; **p<0.01; ***p<0.001. ADL, activities of daily living; ESS, Epworth Sleepiness Scale; HRQoL, health-related quality of life; MCSI, Modified Caregiver Strain Index; NMSS, Non-Motor Symptoms Scale; PDSS-2, Parkinson’s Disease Sleep Scale-2; PDQ-8, 8-item Parkinson’s Disease Questionnaire; SD, standard deviation; UDysRS, Unified Dyskinesia Rating Scale, UPDRS, Unified Parkinson’s Disease Rating Scale.

### Non-motor symptoms

**Fig. 3:**
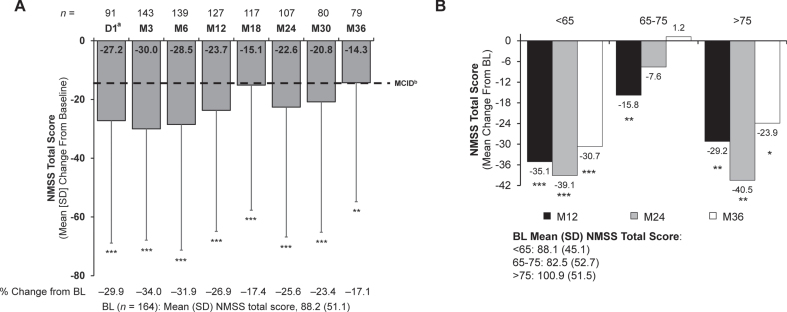
Change from baseline in (A) NMSS total score and (B) NMSS total score by age subgroups. Significance level for change from baseline was determined using the one-sample t test. *p<0.05; **p<0.01; ***p<0.001. ^a^Only patients who participated in the preceding nasojejunal test phase were assessed at D1. ^b^As reported in Martinez-Martin et al [31]. BL, baseline; D, day; M, month; MCID, minimal clinically important difference; NMSS, Non-Motor Symptom Scale; SD, standard deviation.

NMSS total scores demonstrated significant and sustained improvement from baseline at Month 36 (mean [SD], –14.3 [40.5]; *p* = 0.002) and all time points (Fig. 3A,B). In addition, three of nine NMSS subdomains were significantly improved through Month 36: sleep/fatigue (mean [SD], –3.7 [12.3]; *p* = 0.007), gastrointestinal tract (–2.5 [6.1]; *p* < 0.001), and miscellaneous (–3.9 [10.3]; *p* < 0.001) (Supplementary Figure 5). Of note, the last statistically significant improvement was measured at Month 12 for one domain (cardiovascular, including falls), and Month 24 for three domains (mood/cognition, attention/memory, and sexual function) (Supplementary Figure 5).

**Fig. 4:**
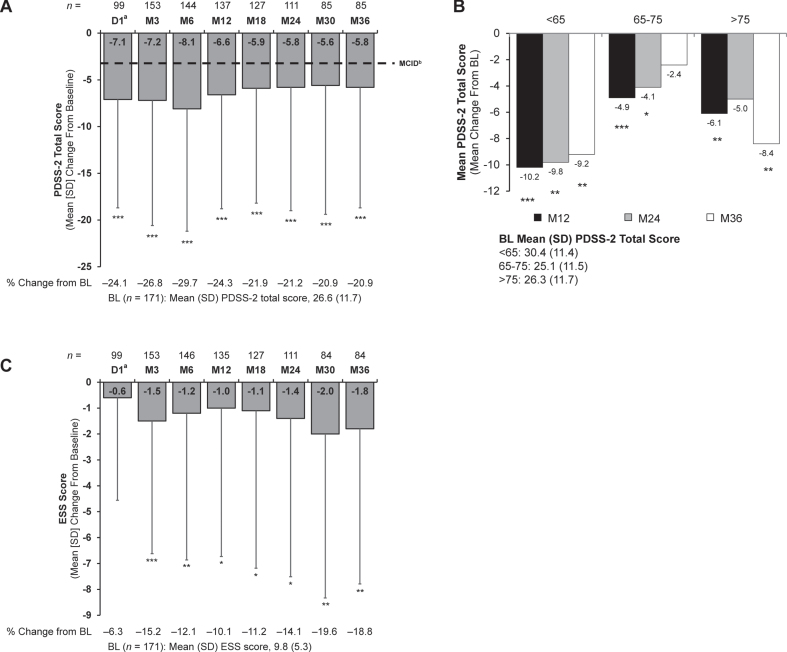
Change from baseline in (A) PDSS-2 total score, (B) PDSS-2 total score by age subgroups, and (C) ESS total score. Significance level for change from baseline was determined using the one-sample *t* test. *p<0.05; **p<0.01; ***p<0.001. ^a^Only patients who participated in the preceding nasojejunal test phase were assessed at D1. ^b^As reported in Horvath et al [32]. BL, baseline; D, day; ESS, Epworth Sleepiness Scale; M, month; MCID, minimal clinically important difference; PDSS-2, Parkinson’s Disease Sleep Scale-2; SD, standard deviation.

In addition to improvement in the NMSS sleep/fatigue subdomain, stable and significant improvement in sleep quality (Fig. 4A,B) and daytime sleepiness (Fig. 4C) was seen with LCIG. Significant improvement from baseline at Month 36 was observed for PDSS-2 total scores (mean [SD], –5.8 [12.9]; *p* < 0.001) and ESS total scores (mean [SD], –1.8 [6.0]; *p* = 0.008). Significant improvement was seen from Day 1 (PDSS-2) and Month 3 (ESS) onward.

### Patient HRQoL and caregiver burden

**Fig. 5:**
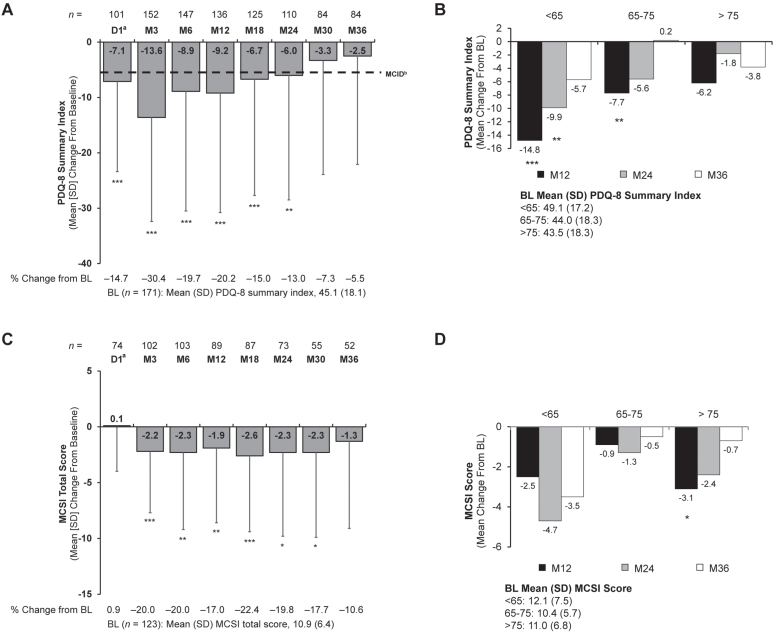
Change from baseline in (A) PDQ-8 summary index, (B) PDQ-8 summary index by age subgroups, (C) MCSI total score, and (D) MCSI total score by age subgroups. Significance level for change from baseline was determined using the one-sample *t* test. *p<0.05; **p<0.01; ***p<0.001. ^a^Only patients who participated in the preceding nasojejunal test phase were assessed at D1. ^b^As reported in Horvath et al [36]. BL, baseline; D, day; MCID, minimal clinically important difference; MCSI, Modified Caregiver Strain Index; PDQ-8, 8-item Parkinson’s Disease Questionnaire; SD, standard deviation.

Patient HRQoL and caregiver burden improved from baseline, with significant and consistent improvement in PDQ-8 through Month 24 (mean [SD]: –6.0 [22.5]; *p* = 0.006) (Fig. 5A,B) and Modified Caregiver Strain Index through Month 30 (–2.3 [7.6]; *p* = 0.026) (Fig. 5C,D). Both scales continued to show numerical, but not statistically significant, improvements at Month 36.

### Safety

A total of 107 (54.9%) patients experienced SAEs (Table 3), with 31 SAEs considered as having a reasonable possibility of being related to LCIG treatment. The most common SAEs were fall (*n* = 8), (worsening of) PD (*n* = 8), and urinary tract infection (*n* = 7) (Table 3). One patient experienced an SAE of polyneuropathy, and one experienced an SAE of chronic inflammatory demyelinating polyradiculoneuropathy; both SAEs were adjudicated by the investigator as having no reasonable possibility of relationship to study drug. A total of 53 (27.2%) patients discontinued the study owing to an SAE. However, this includes 34/195 (17.4%) fatal AEs, with all reported as having no reasonable possibility of relationship to study treatment with the exception of one patient with an intestinal obstruction and a medical history of diverticulitis that was adjudicated as possibly related to treatment (Supplementary Table 1). Most fatal AEs were related to complications of aPD, cardiovascular disease, and complications from non-treatment-related infections.

**Table 3:** Safety summary

Parameters			*n* (% of *N* = 195)
Any SAE			107 (54.9)
Any SAE with reasonable possibility of causal relationship to LCIG			31 (15.9)
Any SAE leading to drug withdrawal			53 (27.2)
Any severe AE			69 (35.4)
Patients remaining on LCIG despite study discontinuation			32 of 106 discontinued
			patients (30.2%)
Deaths			34 (17.4)
Deaths considered possibly related to LCIG^a^			1 (0.5)
		Common SAEs (≥4 patients)	Treatment-emergent SAEs (reasonable possibility)
MedDRA v23.1 Preferred Term		*n* (% of *N* = 195)	*n* (% of *N* = 195)
Fall		8 (4.1)	2 (1.0)
PD		8 (4.1)	3 (1.5)
Urinary tract infection		7 (3.6)	1 (0.5)
Hip fracture		6 (3.1)	0
Pneumonia		6 (3.1)	0
Abdominal pain		6 (3.1)	4 (2.1)
Device dislocation		5 (2.6)	2 (1.0)
Femoral neck fracture		4 (2.1)	0
Hyponatremia		4 (2.1)	1 (0.5)
Sepsis		4 (2.1)	0
Treatment Emergent AEs, *n* (%)	<65 y (*n* = 44)	65–75 y (*n* = 95)	>75 y (*n* = 56)
Any severe AE	13 (29.5)	36 (37.9)	20 (35.7)
Any SAE	21 (47.7)	58 (61.1)	28 (50.0)
Any SAE with reasonable possibility of causal relationship to LCIG	4 (9.1)	16 (16.8)	11 (19.6)
Any SAE leading to drug withdrawal	8 (18.2)	31 (32.6)	14 (25.0)
Deaths	7 (15.9)	19 (20.0)	8 (14.3)
Deaths considered possibly related to LCIG	0	1 (1.1)	0
SAEs in≥4 patients in total group, *n* (%)	<65 y (*n* = 44)	65–75 y (*n* = 95)	>75 y (*n* = 56)
Fall	2 (4.5)	2 (2.1)	4 (7.1)
PD	0	5 (5.3)	3 (5.4)
Urinary tract infection	1 (2.3)	4 (4.2)	2 (3.6)
Hip fracture	0	2 (2.1)	4 (7.1)
Pneumonia	1 (2.3)	4 (4.2)	1 (1.8)
Abdominal pain	1 (2.3)	3 (3.2)	2 (3.6)
Device dislocation	0	3 (3.2)	2 (3.6)
Femoral neck fracture	0	2 (2.1)	2 (3.6)
Hyponatremia	0	3 (3.2)	1 (1.8)
Sepsis	0	1 (1.1)	3 (5.4)

^a^Intestinal obstruction. AE, adverse event; LCIG, levodopa-carbidopa intestinal gel; MedDRA, Medical Dictionary for Regulatory Activities; PD, Parkinson’s disease; SAE, serious adverse event.

A total of six patients (3.1%) discontinued the study due to COVID-related infections, restrictions, or fears of infection. These discontinuations included two patients (1.0%) who had an SAE of COVID-19 and/or COVID-related pneumonia, both of which resulted in death.

In general, body weight and body mass index (BMI) remained relatively stable throughout the study. At baseline, mean body weight was 73.2 kg and mean BMI was 25.7 kg/m^2^; at Month 36, mean weight and BMI were 71.2 kg and 24.9 kg/m^2^, respectively, with both decreasing throughout the study (Supplementary Figure 6A). Mean (SD) decrease from baseline to Month 36 was –3.1 (8.0) kg for weight and –1.2 (2.8) kg/m^2^ for BMI. At last visit, 8.2% of patients demonstrated an increase in baseline weight≥7%, while 22.1% demonstrated≥7% decrease. Most patients remained within the same BMI category at the end of the study that they were in at baseline (Supplementary Figure 6B).

Looking at safety findings across age, patients in the 65- to 75-year age group experienced higher rates of SAEs than did those in the younger than 65-year age group and older than 75-year age groups (whose rates were similar to each other), with rates of severe AEs lowest in the younger than 65-year age group and similar in the 65 to 75 year and older than 75-year age groups (Table 3). Rates of severe AEs were lowest in patients aged younger than 65 years and were similar in patients aged 65 to 75 years and older than 75 years.

## DISCUSSION

We report the final results of DUOGLOBE, a 3-year, fully prospective study designed to evaluate LCIG use in routine clinical practice in a large real-world population with aPD. Treatment with LCIG demonstrated “Off” time improvements that were maintained over 3 years and remained above the minimal clinically important difference (MCID) of 1 hour in this population, despite the progressive nature of PD [26].

The beneficial effects of LCIG use were also reflected in improvements in UDysRS scores and subscores. Improvements in “On” dyskinesia (Part I) were above the MCID of –2.1 through Month 24 and improvements in “Off” dystonia (Part II) were above the MCID of –1.8 through Month 36 [25]. Reductions in UDysRS scores are particularly noteworthy, as this scale (which includes both patient subjective and clinician objective dyskinesia ratings) has demonstrated a high sensitivity for detecting treatment-related changes [27]. The current findings of significant decreases in “Off” time coupled with significant improvements in “On” dyskinesia with LCIG treatment are consistent with those reported in previous studies [15, 28, 29]. Parallel improvements in “Off” time and “On” time with dyskinesia are possible with LCIG, likely due to continuous delivery of stable plasma levodopa levels that can remain in the therapeutic window [10, 15, 28].

Non-motor symptoms of PD are often unrecognized and untreated [30]. Significant improvements were observed in the NMSS total scores, which remained above the 13.9-point MCID throughout the 3 years [31]. Improvements in sleep and daytime sleepiness (as measured by the PDSS-2 and ESS, respectively) were also observed throughout the study, with PDSS-2 changes exceeding the –3.4 point MCID threshold at all timepoints [32]. These findings are particularly relevant, as non-motor symptoms and sleep disturbances have been directly linked to deterioration of HRQoL [33–35]. This study supports this relationship, with both non-motor symptoms and HRQoL improving after LCIG treatment. Significant improvements in the PDQ-8 Summary Index were observed at each time point, with changes at most time points exceeding the MCID threshold of –5.9 [36]. Importantly, while improvements in the NMSS have been documented with LCIG [18, 37], this is the first study to show that specific non-motor symptoms (sleep, gastrointestinal, miscellaneous) continued to show sustained improvement after 3 years of treatment, with cardiovascular symptoms showing significant improvement until Month 12 and mood/cognition, attention/memory, and sexual function showing significant improvement until Month 24.

Patient mood, in particular depression, and patient quality of life are associated with caregiver burden [38]. Previous investigations of the impact of LCIG on caregiver strain have reported conflicting results [12, 20, 39–42], likely as a result of different measurements used and methodologic challenges like different caregivers in longitudinal follow-up studies. Findings from our study indicate that caregiver burden is reduced by patient use of LCIG, signifying that improvements in patient symptoms observed with LCIG use and/or replacement of complex oral dosing with LCIG may reduce strain oncaregivers.

Despite initial improvement, UPDRS Part II and Part III scores significantly worsened by Month 36, likely reflecting underlying disease progression and worsening of motor function in daily living over the 3-year study. This finding is consistent with the finding in a phase 3 open-label trial of LCIG wherein UPDRS total score, Part II, and Part III scores demonstrated significant initial improvements that were not maintained over the course of the 4.1-year follow-up [43]. Interestingly, in the current trial, the dose of LCIG remained relatively stable throughout its 3-year duration, indicating dose adjustments did not need to be made for the progression of disease.

Overall, safety data are consistent with the well-established safety profile of LCIG. Slightly more than half the patients experienced an SAE, with event frequency and type as expected in this 3-year study of a patient population that is of advanced age with multiple comorbidities. Because weight loss is often seen in patients with PD, particularly in the later stages of PD [44], the average weight loss of 2 kg over 3 years is not surprising. Because of the absence of a control group, it is unclear from this study if LCIG contributes to additional weight loss; however, most patients in this study did not experience more than a 7% change in weight.

Although two patients reported a polyneuropathy during the study, no conclusions can be made owing to the observational character of the study as no systematic assessment of blood parameters such as vitamins B1/6/12, folate, or homocysteine and no nerve conduction velocities were performed. Of note, because only SAEs were reported, any report of polyneuropathy that was not an SAE was not captured. Polyneuropathy has been reported in other 1- to 2-year studies of LCIG, as the risk of polyneuropathy may increase with long-term exposure to levodopa [45–47]. Clinical management of patients receiving LCIG over several years may require careful monitoring for neuropathic symptoms.

Many patients who discontinued the study continued LCIG treatment outside the study, indicating the rate of discontinuations over the 3-year study was not necessarily due to an unfavorable benefit/risk profile for LCIG. Approximately half the patients who discontinued the study did so for non-safety-related reasons. When evaluating those discontinuations resulting from SAEs, more than half were fatal AEs, with only one considered possibly related to study drug. Given this is the first fully prospective international study of LCIG with a 3-year follow-up period, it is not surprising that the rate of fatal AEs observed in this study (17.4%) is higher than that reported in other observational studies of LCIG (3% over 1 year and 8–9% over 2 years) [16, 20, 48]. The vast majority of fatal AEs observed here were related to cardiac events, complications from non-treatment-related infections (like pneumonia, sepsis, etc.), neoplasms, and disease progression, and were not considered related to the study drug by investigators. When considered in conjunction with the discontinuations due to non-safety reasons, it appears reassuring that the number of patients who truly discontinued because of an SAE, other than expected age-related deaths, is relatively small. Of note, a post hoc completer analysis, which compared baseline demographics and disease characteristics of patients who completed the first 12 months of study vs. those who did not, found no clear differences between the groups [24].

As the first fully prospective 3-year multinational, real-world study of long-term LCIG treatment, these findings contribute substantially to our understanding of the long-term management of patients with aPD. In addition, the stability in outcomes over time (including robust improvements in dyskinesia) demonstrates the long-term effectiveness of LCIG in a real-world setting as PD progresses. The study also provides long-term safety data, including discontinuations and weight changes over time, as well as the effects of COVID-19 on patient disposition (the study was in its last phase during the first year of the COVID-19 pandemic). However, with only two COVID-19-related deaths and 3% of discontinuations attributed to COVID-19 (both owing to restrictions and fear of visiting study sites), the study was affected by COVID-19 only to a minor degree.

The main limitation of this study is that it was an open-label observational study, which lacked a treatment comparator, so it is difficult to judge symptom deterioration in the context of a progressive disease. Our ability to reach definitive conclusions regarding the causality of AEs such as polyneuropathy was also limited by a lack of assessment of nerve conduction velocities and regularly scheduled laboratory evaluations, including vitamin B1/6/12, folate, homocysteine, and methyl malonic acid. Also, this study excluded patients with mild-to-severe cognitive deficits or dementia (Mini-Mental State Examination score of less than 24) as well as patients who had undergone deep brain stimulation (excluded only in participating countries outside the United States). Excluding these patient populations limits our understanding of LCIG in these groups. The fact that approximately 50% of patients discontinued the study at some point over the 36 months may limit interpretation of the primary endpoint.

Findings from this study demonstrate the significant and sustained clinical value of LCIG in a practical real-world setting across motor fluctuations, dyskinesia, and non-motor symptoms. Results show improvements in HRQoL and reduction in caregiver burden. Of particular interest is that improvements above the MCID in “Off” time, dyskinesia, non-motor symptoms, sleep, and HRQoL were maintained long-term in a population with an otherwise progressive disease [26, 31, 32, 36]. Safety events were consistent with those identified in previous studies of LCIG. Importantly, 30% of patients who discontinued the study continued to use LCIG outside the study, attesting to the benefits of long-term LCIG use in aPD.

## Supplementary Material

Supplementary Material

## References

[1] Olanow CW , Stocchi F (2018) Levodopa: A new look at an old friend. Mov Disord 33, 859–866. 29178365 10.1002/mds.27216

[2] Olanow CW , Obeso JA , Stocchi F (2006) Continuous dopamine-receptor treatment of Parkinson’s disease: Scientific rationale and clinical implications. Lancet Neurol 5, 677–687. 16857573 10.1016/S1474-4422(06)70521-X

[3] Antonini A , Chaudhuri KR , Martinez-Martin P , Odin P (2010) Oral and infusion levodopa-based strategies for managing motor complications in patients with Parkinson’s disease. CNS Drugs 24, 119–129. 20088619 10.2165/11310940-000000000-00000

[4] Kulisevsky J , Luquin MR , Arbelo JM , Burguera JA , Carrillo F , Castro A , Chacón J , García-Ruiz PJ , Lezcano E , Mir P , Martinez-Castrillo JC , Martínez-Torres I , Puente V , Sesar A , Valldeoriola-Serra F , Yañez R (2013) [Advanced Parkinson’sdisease: Clinical characteristics and treatment (part 1)]. Neurologia 28, 503–521. 23856182 10.1016/j.nrl.2013.05.001

[5] Ray Chaudhuri K , Poewe W , Brooks D (2018) Motor and nonmotor complications of levodopa: Phenomenology, risk factors, and imaging features. Mov Disord 33, 909–919. 30134055 10.1002/mds.27386

[6] Chaudhuri KR , Rizos A , Sethi KD (2013) Motor and nonmotor complications in Parkinson’s disease: An argument for continuous drug delivery? J Neural Transm (Vienna) 120, 1305–1320. 23456290 10.1007/s00702-013-0981-5 PMC3751411

[7] Fox SH , Lang AE (2008) Levodopa-related motor complications–phenomenology, Mov Disord 23 Suppl 3, S509–514. 18781677 10.1002/mds.22021

[8] Amjad F , Bhatti D , Davis TL , Oguh O , Pahwa R , Kukreja P , Zamudio J , Metman LV (2019) Current practices for outpatient initiation of levodopa-carbidopa intestinal gel for management of advanced Parkinson’s disease in the United States. Adv Ther 36, 2233–2246. 31278691 10.1007/s12325-019-01014-4 PMC6822848

[9] Zulli C , Sica M , De Micco R , Del Prete A , Amato MR , Tessitore A , Ferraro F , Esposito P (2016) Continuous intra jejunal infusion of levodopa-carbidopa intestinal gel by jejunal extension tube placement through percutaneous endoscopic gastrostomy for patients with advanced Parkinson’s disease: A preliminary study. Eur Rev Med Pharmacol Sci 20, 2413–2417. 27338069

[10] Othman AA , Rosebraugh M , Chatamra K , Locke C , Dutta S (2017) Levodopa-carbidopa intestinal gel pharmacokinetics: Lower variability than oral levodopa-carbidopa. J Parkinsons Dis 7, 275–278. 28211816 10.3233/JPD-161042 PMC5438475

[11] Fernandez HH , Standaert DG , Hauser RA , Lang AE , Fung VS , Klostermann F , Lew MF , Odin P , Steiger M , Yakupov EZ , Chouinard S , Suchowersky O , Dubow J , Hall CM , Chatamra K , Robieson WZ , Benesh JA , Espay AJ (2015) Levodopa-carbidopa intestinal gel in advanced Parkinson’s disease: Final 12-month, open-label results. Mov Disord 30, 500–509. 25545465 10.1002/mds.26123 PMC4674978

[12] Olanow CW , Kieburtz K , Odin P , Espay AJ , Standaert DG , Fernandez HH , Vanagunas A , Othman AA , Widnell KL , Robieson WZ , Pritchett Y , Chatamra K , Benesh J , Lenz RA , Antonini A , Group LHS (2014) Continuous intrajejunal infusion of levodopa-carbidopa intestinal gel for patients with advanced Parkinson’s disease: A randomised, controlled, double-blind, double-dummy study. Lancet Neurol 13, 141–149. 24361112 10.1016/S1474-4422(13)70293-X PMC4643396

[13] Slevin JT , Fernandez HH , Zadikoff C , Hall C , Eaton S , Dubow J , Chatamra K , Benesh J (2015) Long-term safety and maintenance of efficacy of levodopa-carbidopa intestinal gel: An open-label extension of the double-blind pivotal study in advanced Parkinson’s disease patients. J Parkinsons Dis 5, 165–174. 25588353 10.3233/JPD-140456

[14] Standaert DG , Rodriguez RL , Slevin JT , Lobatz M , Eaton S , Chatamra K , Facheris MF , Hall C , Sail K , Jalundhwala YJ , Benesh J (2017) Effect of Levodopa-carbidopa intestinal gel on non-motor symptoms in patients with advanced Parkinson’s disease. Mov Disord Clin Pract 4, 829–837. 29242809 10.1002/mdc3.12526 PMC5724683

[15] Freire-Alvarez E , Kurča E , Lopez Manzanares L , Pekkonen E , Spanaki C , Vanni P , Liu Y , Sánchez-Soliño O , Barbato LM (2021) Levodopa-carbidopa intestinal gel reduces dyskinesia inParkinson’s disease in a randomized trial. Mov Disord 36, 2615–2623. 34236101 10.1002/mds.28703 PMC9292774

[16] Antonini A , Poewe W , Chaudhuri KR , Jech R , Pickut B , Pirtosek Z , Szasz J , Valldeoriola F , Winkler C , Bergmann L , Yegin A , Onuk K , Barch D , Odin P , GLORIA study co-investigators (2017) Levodopa-carbidopa intestinal gel in advanced Parkinson’s: Final results of the GLORIA registry. Parkinsonism Relat Disord 45, 13–20. 29037498 10.1016/j.parkreldis.2017.09.018

[17] Fasano A , Gurevich T , Jech R , Kovács N , Svenningsson P , Szász J , Parra JC , Bergmann L , Johnson A , Sanchez-Soliño O , Tang Z , Vela-Desojo L (2021) Concomitant medication usage withlevodopa-carbidopa intestinal gel: Results from the COSMOS Study. Mov Disord 36, 1853–1862. 33908647 10.1002/mds.28596 PMC8453961

[18] Honig H , Antonini A , Martinez-Martin P , Forgacs I , Faye GC , Fox T , Fox K , Mancini F , Canesi M , Odin P , Chaudhuri KR (2009) Intrajejunal levodopa infusion in Parkinson’s disease: A pilot multicenter study of effects on nonmotor symptoms and quality of life. Mov Disord 24, 1468–1474. 19425079 10.1002/mds.22596

[19] Kamel WA , Al-Hashel JY (2020) LCIG in treatment of non-motor symptoms in advanced Parkinson’s disease: Review of literature. Brain Behav 10, e01757. 32677345 10.1002/brb3.1757 PMC7507541

[20] Lopiano L , Modugno N , Marano P , Sensi M , Meco G , Solla P , Gusmaroli G , Tamma F , Mancini F , Quatrale R , Zangaglia R , Bentivoglio A , Eleopra R , Gualberti G , Melzi G , Antonini A (2019) Motor and non-motor outcomes in patients with advanced Parkinson’s disease treated with levodopa/carbidopa intestinal gel: Final results of the GREENFIELD observational study. J Neurol 266, 2164–2176. 31134377 10.1007/s00415-019-09337-6 PMC6687881

[21] Valldeoriola F , Catalán MJ , Escamilla-Sevilla F , Freire E , Olivares J , Cubo E , García DS , Calopa M , Martínez-Martín P , Parra JC , Arroyo G , Arbelo JM (2021) Patient and caregiver outcomes with levodopa-carbidopa intestinalgel in advanced Parkinson’s disease. NPJ Parkinsons Dis 7, 108. 34848716 10.1038/s41531-021-00246-y PMC8633325

[22] Chaudhuri KR , Antonini A , Pahwa R , Odin P , Titova N , Thakkar S , Snedecor SJ , Hegde S , Alobaidi A , Parra JC , Zadikoff C , Bergmann L , Standaert DG (2022) Effects of levodopa-carbidopa intestinal gel on dyskinesia and non-motor symptoms including sleep: Results from a meta-analysis with 24-month follow-up. J Parkinsons Dis 12, 2071–2083. 35964203 10.3233/JPD-223295 PMC9661331

[23] Antonini A , Odin P , Pahwa R , Aldred J , Alobaidi A , Jalundhwala YJ , Kukreja P , Bergmann L , Inguva S , Bao Y , Chaudhuri KR (2021) The long-term impact of levodopa/carbidopa intestinal gel on ‘off’-time in patients with advanced Parkinson’s disease: A systematic review. Adv Ther 38, 2854–2890. 34018146 10.1007/s12325-021-01747-1 PMC8189983

[24] Standaert DG , Aldred J , Anca-Herschkovitsch M , Bourgeois P , Cubo E , Davis TL , Iansek R , Kovács N , Pontieri FE , Siddiqui MS , Simu M , Bergmann L , Kukreja P , Robieson WZ , Chaudhuri KR (2021) DUOGLOBE:One-year outcomes in a real-world study of levodopa carbidopaintestinal gel for Parkinson’s disease. Mov Disord Clin Pract 8, 1061–1074. 34631942 10.1002/mdc3.13239 PMC8485588

[25] Makkos A , Kovacs M , Pinter D , Janszky J , Kovacs N (2019) Minimal clinically important difference for the historic parts of the Unified Dyskinesia Rating Scale. Parkinsonism Relat Disord 58, 79–82. 30174275 10.1016/j.parkreldis.2018.08.018

[26] Hauser RA , Auinger P , Parkinson Study Group (2011) Determination of minimal clinically important change in early and advanced Parkinson’s disease. Mov Disord 26, 813–818. 21437987 10.1002/mds.23638

[27] Goetz CG , Stebbins GT , Chung KA , Hauser RA , Miyasaki JM , Nicholas AP , Poewe W , Seppi K , Rascol O , Stacy MA , Nutt JG , Tanner CM , Urkowitz A , Jaglin JA , Ge S (2013) Which dyskinesia scale best detects treatment response? Mov Disord 28, 341–346. 23390076 10.1002/mds.25321

[28] Antonini A , Fung VS , Boyd JT , Slevin JT , Hall C , Chatamra K , Eaton S , Benesh JA (2016) Effect of levodopa-carbidopa intestinal gel on dyskinesia in advanced Parkinson’s disease patients. Mov Disord 31, 530–537. 26817533 10.1002/mds.26528 PMC5066747

[29] Poewe W , Bergmann L , Kukreja P , Robieson WZ , Antonini A (2019) Levodopa-carbidopa intestinal gel monotherapy: GLORIA Registry demographics, efficacy, and safety. J Parkinsons Dis 9, 531–541. 31282424 10.3233/JPD-191605 PMC6700622

[30] Lyons KE , Pahwa R (2011) The impact and management of nonmotor symptoms of Parkinson’s disease. Am J Manag Care 17 Suppl 12, S308–314. 22087551

[31] Martinez-Martin P , Rodriguez-Blazquez C , Abe K , Bhattacharyya KB , Bloem BR , Carod-Artal FJ , Prakash R , Esselink RA , Falup-Pecurariu C , Gallardo M , Mir P , Naidu Y , Nicoletti A , Sethi K , Tsuboi Y , van Hilten JJ , Visser M , Zappia M , Chaudhuri KR (2009) International study on the psychometric attributes of the non-motor symptoms scale in Parkinson disease. Neurology 73, 1584–1591. 19901251 10.1212/WNL.0b013e3181c0d416

[32] Horvath K , Aschermann Z , Acs P , Deli G , Janszky J , Komoly S , Karadi K , Kovacs M , Makkos A , Faludi B , Kovacs N (2015) Minimal clinically important difference on Parkinson’s Disease Sleep Scale 2nd Version. Parkinsons Dis 2015, 970534. 26539303 10.1155/2015/970534 PMC4619979

[33] Barone P , Erro R , Picillo M (2017) Quality of life and nonmotor symptoms in Parkinson’s disease. Int Rev Neurobiol 133, 499–516. 28802930 10.1016/bs.irn.2017.05.023

[34] Kovács N , Bergmann L , Anca-Herschkovitsch M , Cubo E , Davis TL , Iansek R , Siddiqui MS , Simu M , Standaert DG , Chaudhuri KR , Bourgeois P , Gao T , Kukreja P , Pontieri FE , Aldred J (2022) Outcomes impactingquality of life in advanced Parkinson’s disease patients treatedwith levodopa-carbidopa intestinal gel. J Parkinsons Dis 12, 917–926. 34974438 10.3233/JPD-212979 PMC9108584

[35] Ray Chaudhuri K , Antonini A , Robieson WZ , Sanchez-Soliño O , Bergmann L , Poewe W, GLORIA study co-investigators (2019) Burden ofnon-motor symptoms in Parkinson’s disease patients predictsimprovement in quality of life during treatment withlevodopa-carbidopa intestinal gel. Eur J Neurol 26, 581–e543. 30353942 10.1111/ene.13847 PMC6590168

[36] Horvath K , Aschermann Z , Kovacs M , Makkos A , Harmat M , Janszky J , Komoly S , Karadi K , Kovacs N (2017) Changes in quality of life in Parkinson’s disease: How large must they be to be relevant? Neuroepidemiology 48, 1–8. 28161701 10.1159/000455863

[37] Politis M , Sauerbier A , Loane C , Pavese N , Martin A , Corcoran B , Brooks DJ , Ray-Chaudhuri K , Piccini P (2017) Sustained striatal dopamine levels following intestinal levodopa infusions in Parkinson’s disease patients. Mov Disord 32, 235–240. 27859651 10.1002/mds.26848

[38] Macchi ZA , Koljack CE , Miyasaki JM , Katz M , Galifianakis N , Prizer LP , Sillau SH , Kluger BM (2020) Patient and caregiver characteristics associated with caregiver burden in Parkinson’s disease: A palliative care approach. Ann Palliat Med 9, S24–S33. 31735048 10.21037/apm.2019.10.01

[39] Caceres-Redondo MT , Carrillo F , Lama MJ , Huertas-Fernandez I , Vargas-Gonzalez L , Carballo M , Mir P (2014) Long-term levodopa/carbidopa intestinal gel in advanced Parkinson’s disease. J Neurol 261, 561–569. 24477490 10.1007/s00415-013-7235-1

[40] Ciurleo R , Corallo F , Bonanno L , Lo Buono V , Di Lorenzo G , Versaci R , Allone C , Palmeri R , Bramanti P , Marino S (2018) Assessment of Duodopa((R)) effects on quality of life of patients with advanced Parkinson’s disease and their caregivers. J Neurol 265, 2005–2014. 29951701 10.1007/s00415-018-8951-3

[41] De Fabregues O , Dot J , Abu-Suboh M , Hernandez-Vara J , Ferre A , Romero O , Ibarria M , Seoane JL , Raguer N , Puiggros C , Gomez MR , Quintana M , Armengol JR , Alvarez-Sabin J (2017) Long-term safety and effectiveness of levodopa-carbidopa intestinal gel infusion. Brain Behav 7, e00758. 28828219 10.1002/brb3.758 PMC5561319

[42] Santos-García D , Añón MJ , Fuster-Sanjurjo L , de laFuente-Fernández R (2012) Duodenal levodopa/carbidopa infusiontherapy in patients with advanced Parkinson’s disease leads toimprovement in caregivers’ stress and burden. Eur J Neurol 19, 1261–1265. 22248261 10.1111/j.1468-1331.2011.03630.x

[43] Fernandez HH , Boyd JT , Fung VSC , Lew MF , Rodriguez RL , Slevin JT , Standaert DG , Zadikoff C , Vanagunas AD , Chatamra K , Eaton S , Facheris MF , Hall C , Robieson WZ , Benesh J , Espay AJ (2018) Long-term safety and efficacy of levodopa-carbidopa intestinal gel in advanced Parkinson’s disease. Mov Disord 33, 928–936. 29570853 10.1002/mds.27338

[44] Uc EY , Struck LK , Rodnitzky RL , Zimmerman B , Dobson J , Evans WJ (2006) Predictors of weight loss in Parkinson’s disease. Mov Disord 21, 930–936. 16534756 10.1002/mds.20837

[45] Zadikoff C , Poewe W , Boyd JT , Bergmann L , Ijacu H , Kukreja P , Robieson WZ , Benesh J , Antonini A (2020) Safety of levodopa-carbidopa intestinal gel treatment in patients with advanced Parkinson’s disease receiving> /=2000mg daily dose of levodopa. Parkinsons Dis 2020, 9716317. 32104560 10.1155/2020/9716317 PMC7040420

[46] Merola A , Romagnolo A , Zibetti M , Bernardini A , Cocito D , Lopiano L (2016) Peripheral neuropathy associated with levodopa-carbidopa intestinal infusion: A long-term prospective assessment. Eur J Neurol 23, 501–509. 26498913 10.1111/ene.12846

[47] Ceravolo R , Cossu G , Bandettini di Poggio M , Santoro L , Barone P , Zibetti M , Frosini D , Nicoletti V , Manganelli F , Iodice R , Picillo M , Merola A , Lopiano L , Paribello A , Manca D , Melis M , Marchese R , Borelli P , Mereu A , Contu P , Abbruzzese G , Bonuccelli U (2013) Neuropathy and levodopa in Parkinson’s disease: Evidence from a multicenter study. Mov Disord 28, 1391–1397. 23836370 10.1002/mds.25585

[48] Krüger R , Lingor P , Doskas T , Henselmans JML , Danielsen EH , de Fabregues O , Stefani A , Sensken SC , Parra JC , Onuk K , Yegin A , Antonini A (2017) An observational study of the effect of levodopa-carbidopa intestinal gel on activities of daily living and quality of life in advanced Parkinson’s disease patients. Adv Ther 34, 1741–1752. 28631218 10.1007/s12325-017-0571-2 PMC5504221

