# Supplementary material for: Levodopa Carbidopa Intestinal Gel in Advanced Parkinson’s Disease: DUOGLOBE Final 3-Year Results
Source: J Parkinsons Dis. 2023 Jul 25;13(5):769–83. doi: 10.3233/JPD-225105 (PMC10473130; doi:10.3233/JPD-225105)
Supplement: Supplementary Material [file jpd-13-jpd225105-s001.pdf]

Supplementary Material

Levodopa Carbidopa Intestinal Gel in Advanced Parkinson's Disease: DUOGLOBE Final 3-Year Results

Supplementary Table 1. Fatal Adverse Events

MedDRA v23.1 Preferred Term	Reasonable possibility of relationship with study drug[a]
Starvation	No
Asphyxia	No
Sudden death	No
Fall	No
Head injury	No
Cerebral infarction	No
Syncope	No
COVID-19	No
Pneumonia	No
Cardiac failure congestive	No
Cardio-respiratory arrest	No
Pneumonia aspiration	No
Dysphagia	No
Failure to thrive	No
Parkinson's disease	No
Hanging	No
Completed suicide	No
Asphyxia	No
Cardiac failure	No
General physical health deterioration	No
Bacterial sepsis	No
Decubitus ulcer	No
Sepsis	No
Pneumonia aspiration	No
Small intestinal obstruction	No
Hemiparesis	No
Death	No
Cardiac arrest	No
Respiratory tract infection	No
Death	No
Cardio-respiratory arrest	No
Clostridium difficile infection	No
Cardiac arrest	No
Death	No
Intestinal obstruction	Yes
Malignant peritoneal neoplasm	No
Death	No
Large intestinal obstruction	No

Volvulus	No
Sepsis	No
COVID-19 pneumonia	No
Lower respiratory tract infection	No
Myocardial infarction	No
B-cell lymphoma stage IV	No
Cardio-respiratory arrest	No

[a]As assessed by the investigator.

Shading indicates grouping by patient.

MedDRA, Medical Dictionary for Regulatory Activities.

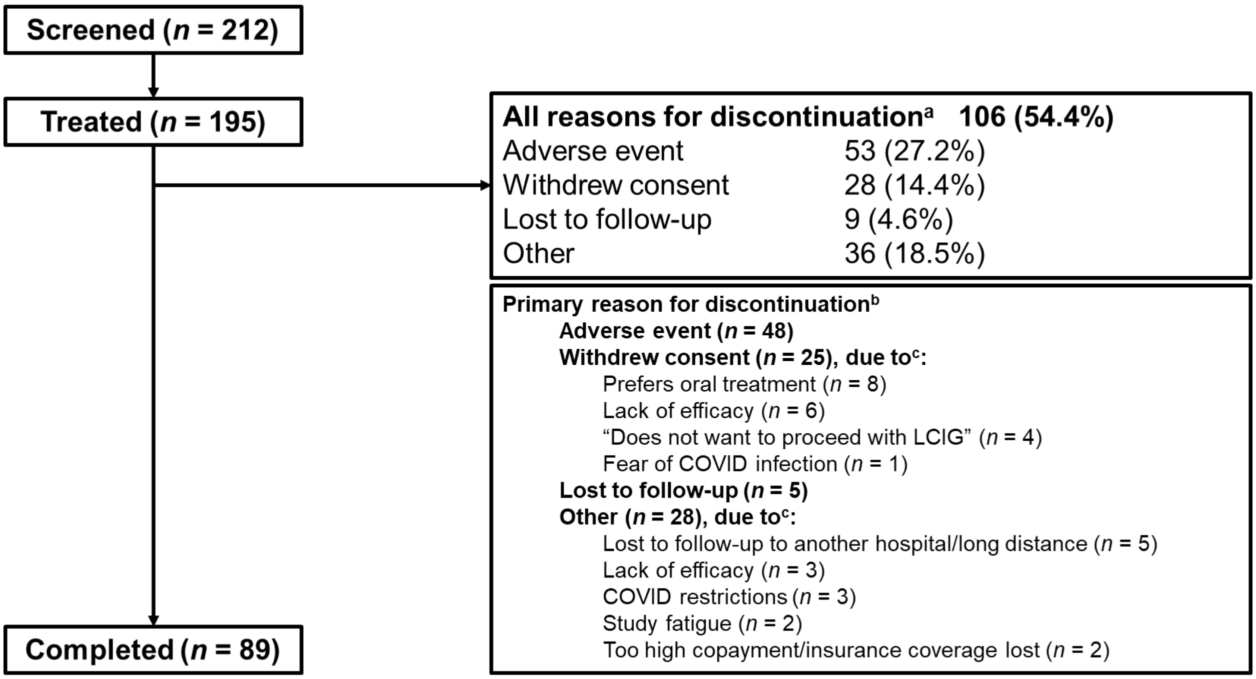

Supplementary Figure 1. Patient disposition.
[a]A total of 32/106 (30.2%) patients who discontinued the study continued LCIG treatment outside the study. [b]Each patient could have multiple reasons for discontinuation. [c]Reasons were investigator-reported. Aside from COVID-related reasons (which was an area of special interest), only reasons with an $n \geq 2$ are listed. LCIG, levodopa-carbidopa intestinal gel.

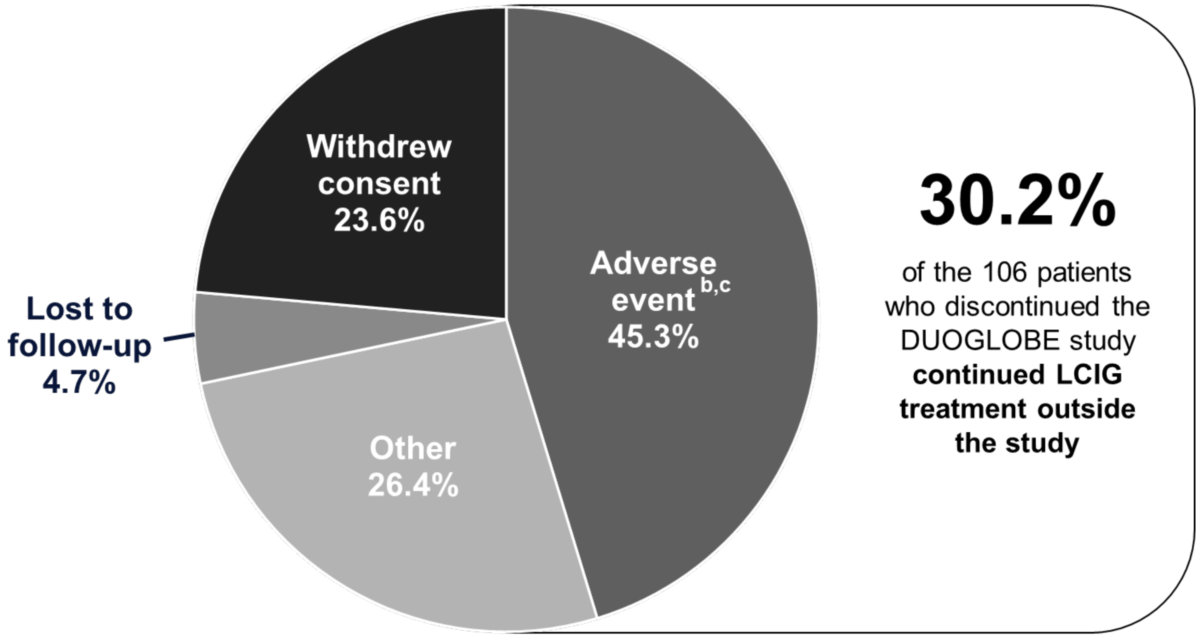

Supplementary Figure 2. Percentage of study discontinuations by primary reason.
[a] Patients could have multiple reasons for discontinuation. Reasons for discontinuation are presented as a proportion of the 106 total study discontinuations. [b]In total, 53 patients reported adverse events leading to discontinuation. Of these, 10 patients reported SAEs that were considered possibly related to the study drug. [c]Includes 34 fatalities, one of which was possibly related to study drug (intestinal obstruction). DUOGLOBE, DUOdopa/Duopa in Patients with Advanced Parkinson's Disease – a GLobal OBservational Study Evaluating Long-Term Effectiveness; LCIG, levodopa-carbidopa intestinal gel; SAE, serious adverse event.

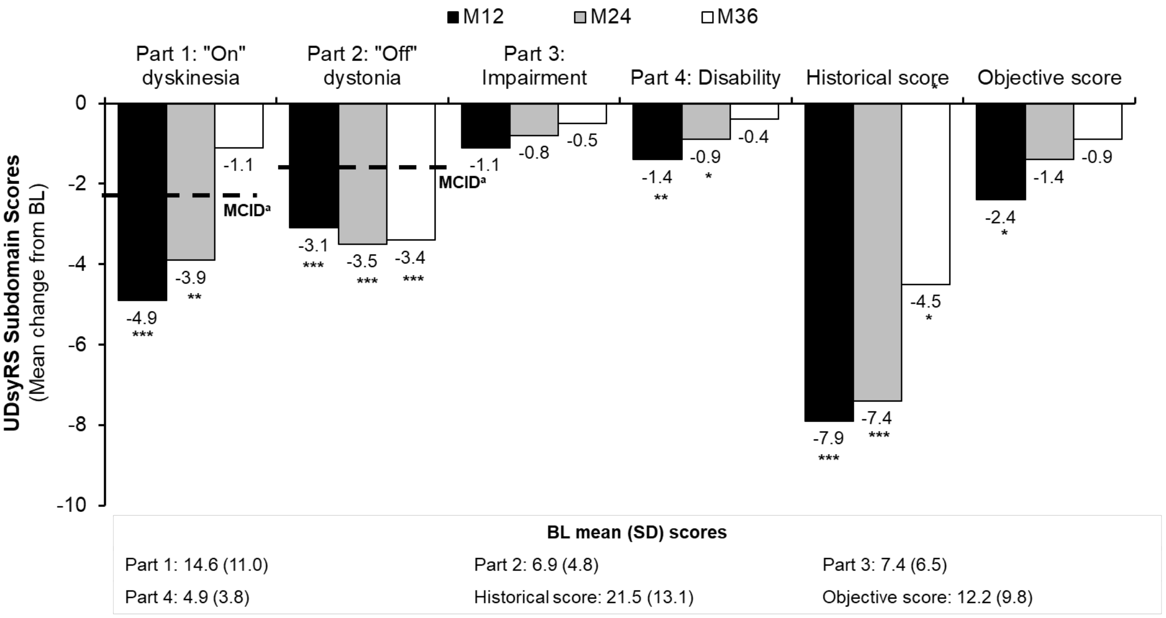

Supplementary Figure 3. Change from baseline in UDysRS subdomain scores. Significance level for change from baseline was determined using the one-sample *t* test. [a]As reported in Makkos et al [25]. *$p < 0.05$; **$p < 0.01$; ***$p < 0.001$. BL, baseline; M, month; MCID, minimal clinically important difference; SD, standard deviation; UDysRS, Unified Dyskinesia Rating Scale.

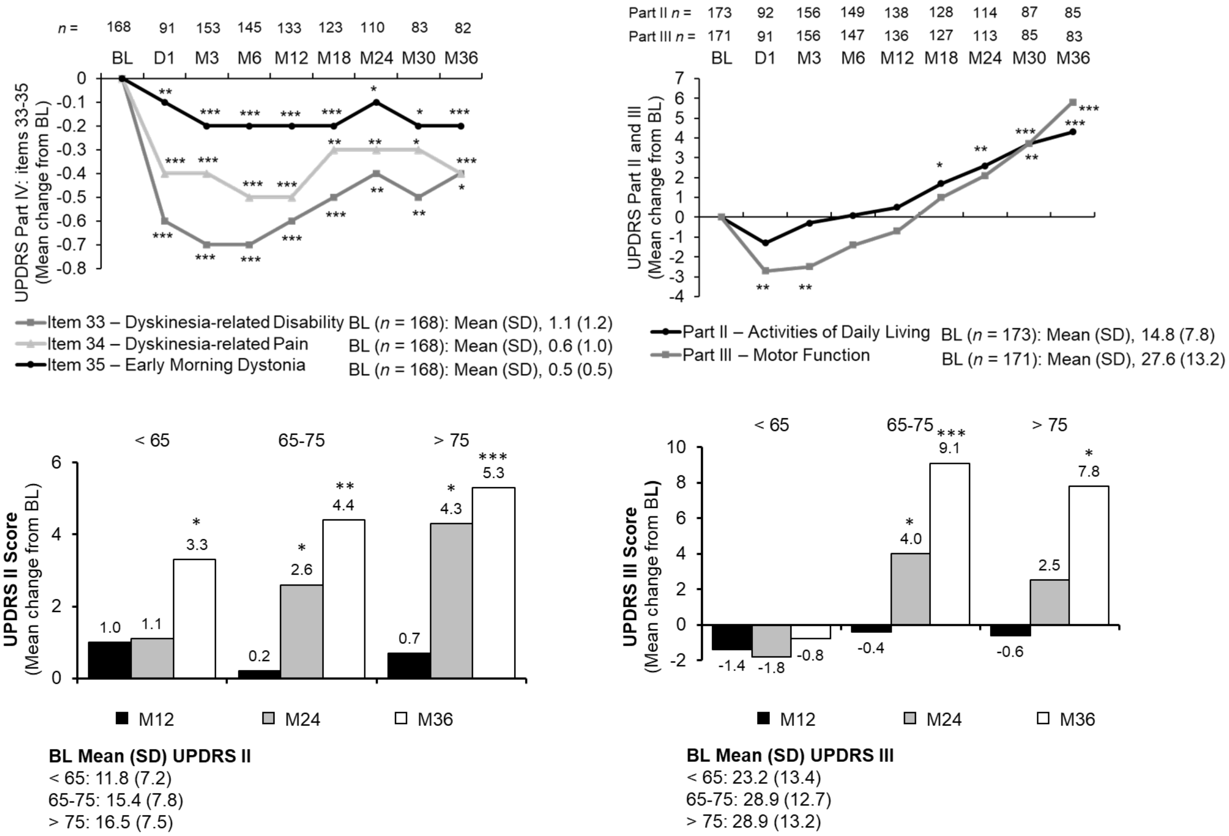

Supplementary Figure 4. Change from baseline in dyskinesia-related UPDRS IV subitems, UPDRS II and III scores.

Significance level for change from baseline was determined using the one-sample *t* test. **p* < 0.05; ***p* < 0.01; ****p* < 0.001. BL, baseline; D, day; M, month; SD, standard deviation; UPDRS, Unified Parkinson's Disease Rating Scale.

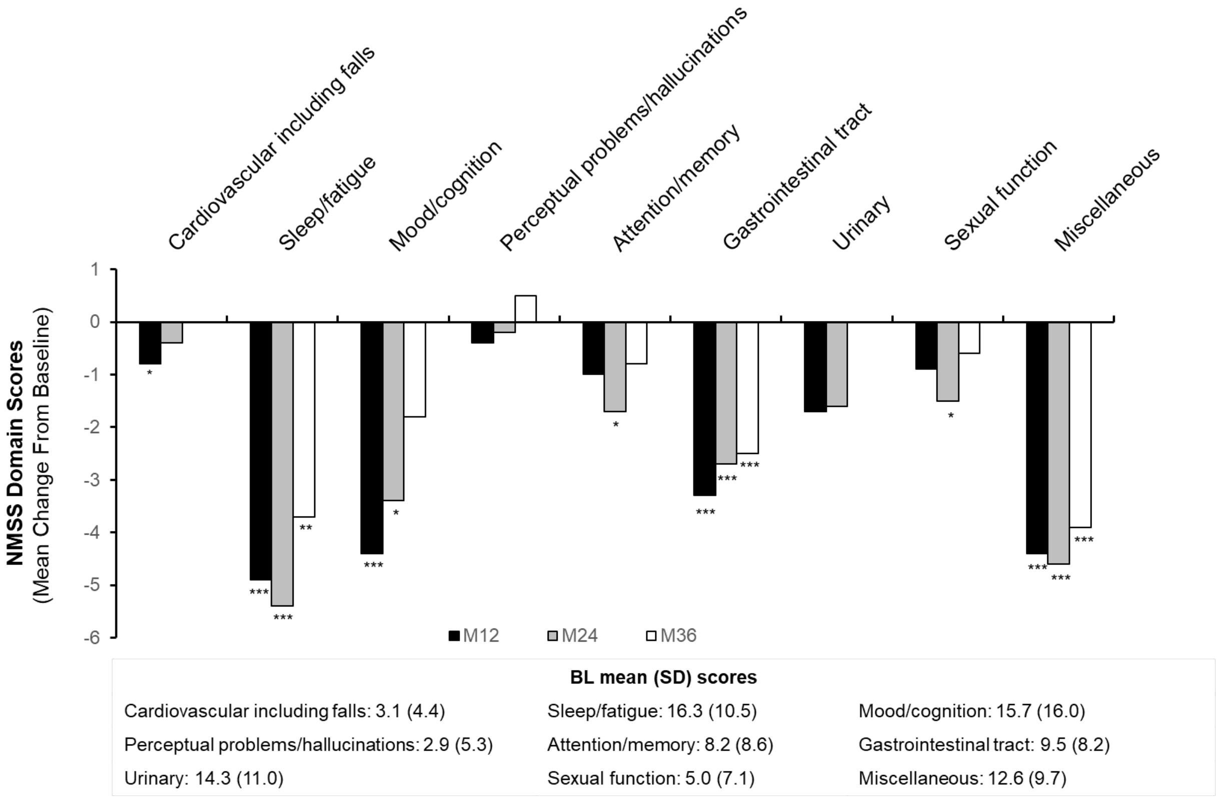

Supplementary Figure 5. Change from baseline in NMSS subdomain scores. Significance level for change from baseline was determined using the one-sample *t* test. *$p < 0.05$; **$p < 0.01$; ***$p < 0.001$. BL, baseline; M, month; NMSS, Non-Motor Symptom Scale.

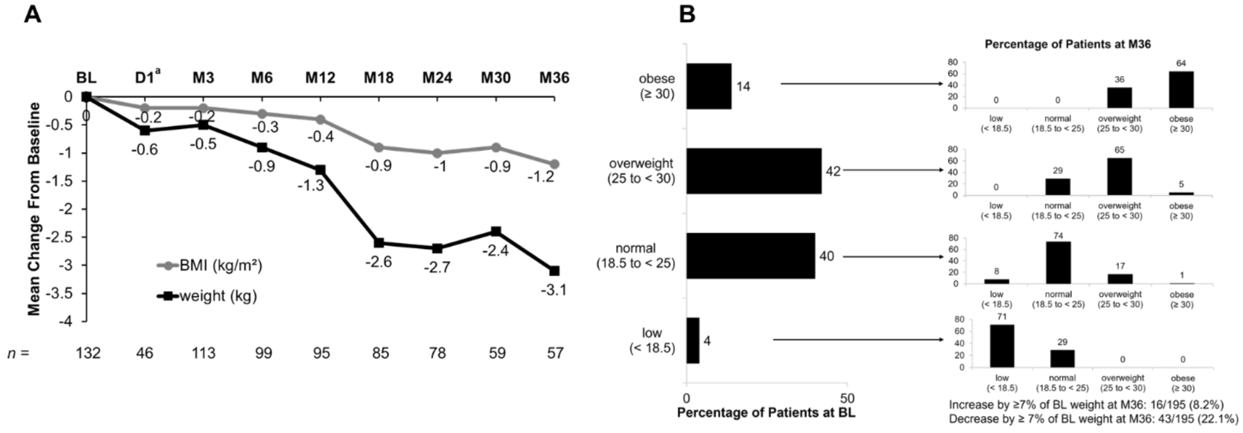

Supplementary Figure 6. Weight and BMI changes (*N* = 195).
(A) Mean change from baseline in weight and BMI and (B) change in BMI categories from baseline to Month 36. [a]Only patients who participated in the nasojejunal test phase were assessed at D1. BL, baseline; BMI, body mass index; D, day; M, month.
